# Screening for infectious diseases of asylum seekers upon arrival: the necessity of the moral principle of reciprocity

**DOI:** 10.1186/s12910-018-0256-7

**Published:** 2018-03-02

**Authors:** Dorien T. Beeres, Darren Cornish, Machiel Vonk, Sofanne J. Ravensbergen, Els L. M. Maeckelberghe, Pieter Boele Van Hensbroek, Ymkje Stienstra

**Affiliations:** 1 Infectious Disease Unit, Department of Internal Medicine, University Medical Centre Groningen, University of Groningen, Groningen, The Netherlands; 2 Babylon Primary Health Care Services, Elst, Groningen, The Netherlands; 3 Department of Infectious Diseases, Regional Public Health Service Groningen, Groningen, The Netherlands; 4 Institute for Medical Education, University Medical Centre Groningen, University of Groningen, Groningen, The Netherlands; 5 Faculty of Philosophy, Department of Ethics, Social and Political Philosophy, University of Groningen, Groningen, The Netherlands

**Keywords:** Reciprocity, Screening, Migration, Infectious diseases, Asylum seeker

## Abstract

**Background:**

With a large number of forcibly displaced people seeking safety, the EU is facing a challenge in maintaining solidarity. Europe has seen millions of asylum seekers crossing European borders, the largest number of asylum seekers since the second world war. Endemic diseases and often failing health systems in their countries of origin, and arduous conditions during transit, raise questions around how to meet the health needs of this vulnerable population on arrival in terms of screening, vaccination, and access to timely and appropriate statutory health services. This paper explores the potential role of the principle of reciprocity, defined as the disposition ‘*to return good in proportion to the good we receive, and to make reparations for the harm we have done’,* as a mid-level principle in infectious disease screening policies.

**Main text:**

More than half of the European countries implemented screening programmes for newly arrived asylum seekers. Screening may serve to avoid potential infectious disease risks in the receiving countries as well as help identify health needs of asylum seekers. But screening may infringe upon basic rights of those screened, thus creating an ethical dilemma.

The use of the principle of reciprocity can contribute to the identification of potential improvements for current screening programmes and emphasizes the importance of certain rights into guidelines for screening. It may create a two way moral obligation, upon asylum seekers to actively participate in the programme, and upon authorities to reciprocate the asylum seekers’ participation and the benefits for the control of public health.

**Conclusion:**

The authors argue that the reciprocity principle leads to a stronger ethical justification of screening programmes and help achieve a balance between justifiable rights claims of the host population and the asylum seekers. The principle deserves a further and more thorough exploration of its potential use in the field of screening, migration and infectious diseases.

## Background

In 2016, the number of forcibly displaced people rose worldwide to 60 million [1]. More than 1.2 million people have crossed the Mediterranean Sea to Europe between January 2015 and May 2016 [2]. Arduous conditions during flight routes and endemic diseases in their countries of origin lead to questions on how to identify and meet the health needs of this vulnerable population.

More than half of the European countries reported to have implemented screening programmes that target newly arrived asylum seekers in 2014 [3]. Screening^1^ for infectious diseases aims to serve two different goals, namely to protect public health by identifying those considered to be a potential source of spreading an infectious disease, and to identify individuals who may benefit from health care so that they can be offered such care [4]. In 2014, a questionnaire-based study described current screening activities for infectious diseases among newly arrived migrants in EU/EEA countries [3]. Of the 27 EEA/EU countries, 15 implemented screenings among newly arrived asylum seekers. Most common screening programmes are for tuberculosis (15/15), Hepatitis B (5/15), HIV (4/15), and Hepatitis C (4/15) [3]. The World Health Organization (WHO) does not recommend compulsory mass screening of the asylum seeker population for infectious diseases because there is no clear evidence proving the effectiveness of screening. Furthermore, screening may create anxiety and reluctance to seek care because asylum seekers may fear that positive test results influence their application procedure [5, 6]. Instead, the WHO and the European Centre for Disease Prevention and Control (ECDC) advise European countries to provide general health checks and improve sanitation and vaccination programmes to prevent outbreaks of infections like measles and influenza [7].

The implementation of various screening programmes and the variation in national guidelines on screening evoke questions about normative justification of such programmes. Policies on screening are not exclusively dependent on the relative risk of infection, but also on non-medical grounds, such as economic, political and emotional ones [2, 4]. Incidences of infectious diseases are often misinterpreted by politicians and the press as a threat. However, epidemiological studies show that this risk is substantially lower than often perceived [2, 5]. While comprehensive data on infectious disease risks are not yet available, it has been shown that some sub-groups of asylum seekers are more affected by several infectious diseases (HIV, tuberculosis and Chagas disease) as compared to the host population [8]. However, there is only a limited risk of disease transmission to the host-population [8]. It is of normative relevance to discuss the proper balance between the state’s obligations to secure public health for all and at the same time to protect the individual rights and needs of asylum seekers.

For assessing these tensions, thoughtful evaluation of moral dilemmas and the values involved is essential. Up to now, the ‘harm principle’ is often mentioned as mid-level normative principle^2^ for addressing the described dilemma in public health. In modern states, even in liberal ones, the obligation to protect the public from serious harm is a foundational principle of public health ethics, and often considered the most compelling justification for public health policies that interfere with individual liberty [10–12]. However, the principle of reciprocity has gained increased attention during the past years. Reciprocity is generally formulated as the disposition “*to return good in proportion to the good we receive, and to make reparations for the harm we have done”* (Becker 1986 p.3) and is considered an important requirement for ethically acceptable public health interventions and screening in a liberal society [13–15]. This principle can be used as a guiding principle in public health issues that are challenged to both fulfil public health duties and at the same time serve individual interests [15, 16].

The aim of this paper is to critically assess the potential role of reciprocity as normative decision and justification principle for screening programmes for asylum seekers. Normative decisions are decisions based on philosophical moral theories on what is valuable (i.e, “what we ought to do”), and moral justification is based on the values set by the related normative principle. The first part of this paper discusses the principle of reciprocity and the way it can be used. The second part evaluates the current practice of screening programmes at reception centres in Europe in the light of reciprocity, thereby assessing the potential of the principle of reciprocity for evaluating the ethical dimension of such screening programmes.

### The principle of reciprocity as a mid-level principle for infectious disease control

The implementation of screening often results in conflicting interests and creates a tension between the obligation of the state to protect the health of the public on one hand, and respecting the autonomy, health interests, and liberty of the screened individuals on the other [16].

The concept of reciprocity may be a helpful notion in analysing such tensions. Literature in the social sciences shows that reciprocity increases the willingness for social cooperation [17]. In public health ethics there is an increasing interest in using reciprocity as a mid-level, action-guiding principle [15]. For instance, the principle is used as instrument to legitimize and justify limitations on human rights in public health emergencies that require restrictive measurements [18]. The SARS outbreak in 2004, the recent Ebola outbreak in 2014, and the continuing discussions around tuberculosis treatment and control have led to some clear formulations of reciprocity in public health ethics [16, 19–21].

While the principle of reciprocity has been formulated in different contexts, virtue theorist Lawrence Becker articulated the most well-known classical formulation of reciprocity as the disposition “*to return good in proportion to the good we receive, and to make reparations for the harm we have done”* (Becker 1986, 3). Viens and colleagues formulated this conception of reciprocity for public health ethics:

“Reciprocity demands an appropriate balancing of the benefits and burdens of social cooperation necessary to obtain the good of public health. (…) Reciprocity requires that we compensate those disproportionately burdened by complying with restrictive measures and make restitution to those individuals wronged by being subjected to unfair or intolerable treatment. Reciprocity not only requires that individuals should not be overly burdened by measures to protect public health, but also that individuals are supported in a way that allows them to fulfil their obligations” (Viens, Bensimon, and Upshur 2009, ref [21], page 211−212).

Seven years later, Silva, Dawson, and Upshur build on this formulation towards a more general definition as:

*“Reciprocity is a moral obligation involving an appropriate response by B commensurate with an action by A, where A’s action aims to contribute to, or bring about, public good, and where the action involves burdens, costs, or risks of harm to A” (Silva, Dawson, and Upshur 2016, ref* [15]*, page 84).*

This definition includes several key features that can be formulated in the context of infectious disease screening as: First, that the asylum seeker (A) complies to active participation in the programme with the aim to identify presence or absence of disease (i.e., contribution to public good). Second, the authorities organizing the screening (B) have an obligation to respond well to the asylum seekers participation in the screening and consequently the test results of the person being screened. This may include, among other things, explanation of the procedure, explanation of consequences of positive and negative test results, and providing accessible treatment.

In addition to its direct goods and harms to the involved parties, the reciprocity principle has also indirect beneficial influences [15]. Indirect moral power can be defined as a person or institution contributing to a system, e.g. the screening programme, without necessarily receiving direct individual benefit or observing others receiving benefit. Acting upon this principle is based on a more general normative value of social cooperation for the public good. Reciprocity generates strong bonds of solidarity among groups, and has proven to be more effective than placing the burdens and benefits upon a small group of individuals [18, 22, 23]. Moreover, reciprocity as an indirect moral power can be considered a form of (social) justice, where inequalities among asylum seekers and host populations in general create duties towards each other [15].

#### General criteria for screening programmes

While clear international guidelines for screening programmes for infectious diseases at reception centres are lacking, general guidelines for screening are available. In contrast to other national screening programmes that target specific groups (e.g. cervical and breast cancer screening programmes), the case of asylum seeker screening is more complex. The timing of these screening programmes as part of the asylum procedure may lead to ideas that the outcomes may influence their asylum procedure. The frequently limited access to health care makes it challenging for health care providers to meet refugees’ health needs. In addition, a wide cultural variation within asylum seeker populations requires a screening programme with extra attention for cultural sensitive care [24]. Internationally, screening programmes must be in accordance with a regulative framework provided by the WHO. These classical screening criteria were established by Wilson and Jungner in 1968 (Table 1). In 2008, the WHO added additional requirements (Table 2) in response to technical and medical advancements [25].

**Table 1:** Wilson and Jungner screening criteria [40]

1. The condition sought should be an important health problem.	
2. There should be an accepted treatment for patients with recognized disease.	
3. Facilities for diagnosis and treatment should be available.	
4. There should be a recognizable latent or early symptomatic stage.	
5. There should be a suitable test or examination.	
6. The test should be acceptable to the population.	
7. The natural history of the condition, including development from latent to declared disease, should be adequately understood.	
8. There should be an agreed policy on whom to treat as patients.	
9. The cost of case-finding (including diagnosis and treatment of patients diagnosed) should be economically balanced in relation to possible expenditure on medical care as a whole.	
10. Case-finding should be a continuing process and not a “once and for all” project

**Table 2:** Synthesis of emerging screening criteria proposed over the past 40 years [25]

1. The screening programme should respond to a recognized need.	
2. The objectives of screening should be defined at the outset.	
3. There should be a defined target population.	
4. There should be scientific evidence of screening programme effectiveness.	
5. The programme should integrate education, testing, clinical services and programme management.	
6. There should be quality assurance, with mechanisms to minimize potential risks of screening.	
7. The programme should ensure informed choice, confidentiality and respect for autonomy.	
8. The programme should promote equity and access to screening for the entire target population.	
9. Programme evaluation should be planned from the outset.	
10. The overall benefits of screening should outweigh the harm.	

These criteria require a programme to focus on important health issues, produce benefits to the public while minimizing harm to the individual, fairly distributing benefits and burdens, and the intervention in the programme must be proportional [25]. Moreover, the requirement to enforce participation in the screening must be proportionate in view of the public health emergency. Although the WHO guidelines effectively set a high standard for screening, the principle of reciprocity prioritizes several aspects that are not explicitly addressed in the WHO criteria. The principle goes beyond requirements on the quality and appropriateness of the screening. Authorities will be urged to reciprocate participation in screening with assisting the individual (or the community) in the fulfilment of their health care needs, including identification of personal health needs and providing accessible treatment when needed [26].

### Role of reciprocity in screening programmes for infectious diseases that target asylum seekers

The final section of this paper assesses the possible role of reciprocity in the existing screening programmes. Currently, a few studies are available which describe practical experiences with infectious disease screening of asylum seekers [6, 27–29]. While there is an increasing interest in studies describing asylum seekers’ experiences, none of them have extensively addressed the role of normative principles in decision-making, justification, or evaluation of these specific screening programmes. The complex character of asylum seeker screening that is mentioned in the previous paragraph, such as their refugee status and cultural differences, shows the necessity to examine the moral underpinnings of these programmes.

The literature on current screening programmes in Europe shows two ‘levels’ of shortcomings; On policy level regarding the limited scope of the current screening programmes and shortcomings that concern practical issues related to the implementation of such programmes. The most extensive studies concern tuberculosis screening in the UK and the overall health assessments in Sweden. These British and Swedish programmes can help to reflect on other programmes in Europe, and comparing these two distinctive programmes reveals common as well as programme specific challenges. The mandatory tuberculosis screening as in the UK is implemented in several European countries such as in Belgium, Germany, the Netherlands, and Switzerland [30].

#### The examples of the UK and Sweden

In the UK, medical examination may be required for any person who is neither a national of the European Economic Area (EEA) or a UK citizen. Medical examination can take place abroad (as part of visa application), upon arrival at a port of entry, or after entry. All persons arriving from countries with a high incidence of tuberculosis are required to undergo medical screening for the active form of tuberculosis. However, usually screening is only performed among persons who seek to remain in the UK for more than six months [31, 32]. For persons who are clearly unwell, a medical examination can be required. If there is a medical condition that has impact upon applicants’ ability to maintain themselves, or if an applicant represents a risk to the national public health, entry to the UK can be refused [31, 32].

Studies addressing experiences with entry screening for tuberculosis frequently report language barriers, cultural differences, fear for discrimination, and stigmatization. In addition, there is a lack of evidence for the effectiveness and efficiency of the screening [2, 6, 27, 28, 33–35]. A study by Seedat et al. (2014) interviewed UK immigrant community health-care leads representing new migrant groups (refugees, asylum seekers and foreign-born individuals that recently arrived in the UK and came from high prevalence disease countries) in London about their views on screening of migrants. This study observed that the current tuberculosis screening in the UK is often perceived to be migrant unfriendly. The study describes concerns among migrants about cultural insensitivity within the services, inhospitable and unfriendly experiences when accessing the services, and discrimination by health-care professionals [6]. Migrants prefer a general health check-up instead of limited tuberculosis screening. They believe that this will better meet their health needs and reduce stigma [6].

How can the principle of reciprocity guide the British programme? The principle suggests that the benefit of identification of infected individuals to prevent outbreaks creates the reciprocal moral obligation of the authorities to identify other health needs of the asylum seeker apart from tuberculosis and guide them to proper treatment opportunities. Reports of the WHO observe that asylum seekers in general have health problems similar to the host population of receiving countries [5]. To illustrate, the tuberculosis prevalence in Syria is substantially lower than the tuberculosis prevalence in European countries like Bulgaria, Portugal, Poland, and Romania [5, 36]. However, the epidemiological background and the arduous journey many asylum seekers have endured, might increase the risk for mental health problems and the vulnerability to non-communicable (e.g. travel injuries, hypertension) as well as communicable diseases [2, 5]. Asylum seekers are likely to benefit from early diagnosis and treatment, which suggests screening and treatment within the first six months after arrival [36]. Further studies are needed to investigate the best timing of screening. Finally, adequate assessment of immunisation status is important among asylum seeker populations originating from countries with interrupted vaccination programmes.

Summarizing, the present tuberculosis screening programme can be used as a starting point for further mental and physical examination which gives the opportunity for health care interventions for asylum seekers at an early stage. Such an approach, following Molms’ theory on the power of reciprocity in social cooperation, will stimulate asylum seekers to actively participate in these programmes [17].

#### Health assessments in Sweden

While in most European countries disease screening is limited to tuberculosis screening, in Sweden, the National Board of Health and Welfare (NHWB) offer adult and child migrants health screening soon after arrival in accordance with the Act (2008:344) on Health Care for Asylum Seekers and ‘Others’, and the Communicable Disease Act (2004:168) [27]. The health screening is performed soon after arrival, by local health services and is free of charge. The screening includes: an interview about the newcomer’s past and current physical and mental health, vaccination status, and information that may be needed from infection control standpoint. A physical examination and blood test may be performed if necessary [27]. While the health screening in Sweden is voluntary, it can be compulsory under the Communicable Disease Act [37].

The study by Nkulu Kalengayi et al. (2016) describes asylum seekers’ experiences with the health screening in Sweden and shows that the asylum seekers acknowledge that the screening is both beneficial for the host society and for themselves - being grateful to be given the opportunity to have their health status checked. However, they stated that the screening did not meet their expectations. They experienced the screening to be mainly focused on the identification of infectious diseases instead of identification of their health needs [27]. Additionally, the study describes a major concern among asylum seekers that screening outcomes negatively influence their residency. This concern about residency is described to be more important to them than identification of their health needs [27]. It is important to take this finding into account in future strategies given the fact that the timing of screening could have large impact on the uptake of these programmes.

#### Limitations on the principle

While this paper advocates for a directive role of the principle of reciprocity in health policies, there are challenges. A possible objection against the use of reciprocity as a guiding principle in the context of infectious disease screening of asylum seekers could be to argue that the primary goal of active participation of the asylum seeker is not serving public good, but to serve individual benefit (identification of the most important heath needs of the asylum seeker). This assumption may exempt authorities from a moral obligation to compensate for the public health benefits received since the benefits of screening initially serve the individual. However, this objection may be countered by stating that screening solely focused on infectious diseases ignores non-communicable diseases such as mental health problems that pose a bigger threat than communicable diseases on asylum seekers [5, 37].

Second, the obligations and responsibilities that are discussed in the article come from an ethical background, describing actions as they ‘ought to be’. Practical experiences with screening, such as arguments on cost-effectiveness of screening programmes, could create a tension with the arguments derived from the reciprocity principle. While hepatitis screening could be cost-effective on the long-term [38], implementation of screening programmes may result in financial problems on short-term [39]. Increased case identification as a result of the programme could possibly lead to an enormous rise in treatment costs, where the burden on the national health care coverage leads to more harm than the benefits received for the individuals being screened. In these circumstances, moral obligations may describe needs or require resources that could not be met. Re-evaluation of moral responsibilities by balancing benefits and costs is important.

Finally, it is a challenge to identify the best practice that is needed to create an environment that invites both parties to reciprocate to the greatest degree and fulfil their moral obligations. As described in the Swedish study, concerns about residency among asylum seekers and fear for authorities negatively influences active participation among asylum seekers in screening programmes. Given that this could have a large impact on the uptake and efficacy of screening, it is important to further investigate best practice. Similarly, European countries face responsibilities towards each other to cooperate on screening programmes and health policies in the light of reciprocity. Cooperation is important to avoid unnecessary replication of diagnostics and treatment and to avoid country specific preferences among asylum seekers based on differences in screening and health services on arrival, which may result in an unbalanced burden on several countries. Both timing and international agreements are needed to effectively balance moral obligations of both parties and invite host countries to provide best services.

## Conclusions

Reciprocity goes beyond prevention of harm. The current WHO screening guidelines create obligations for authorities to guarantee the quality of screening programmes, but the inclusion of moral obligations is limited. The use of the reciprocity principle could effectively mend this limitation, and deserves a further and more thorough exploration of its potential use in the field of screening, migration and infectious diseases.

The principle identifies limitations in current screening programmes and emphasizes the importance of certain rights (e.g. autonomy, right to health) into guidelines for screening. It creates a moral obligation upon authorities to reciprocate the asylum seekers’ forced participation and the benefits for the control of public health. The attention to the reciprocity norm suggests two ‘levels’ at which current screening programmes can be improved.

Screening is made beneficial for the asylum seekers if it is not limited to only infectious diseases identification, but takes a more holistic approach by focussing on mental and physical health problems, including vaccination and prevention programmes and accessible information about the national healthcare system [27, 35]. Second, the screening process requires that the benefits for the receiving society and the obligation for the asylum seekers to respond and actively participate in the programme are balanced with health benefits of those screened, who see the screening followed-up by adequate general health care.

## Footnotes

1 In the context of this article screening means the systematic application of a medical test to a defined population (e.g. asylum seekers) with the purpose to identify those with undetected disease.

2 Moral mid-level principles are moral principles in which their application is specialized within a specific field. They stand in between unconditional principles (e.g. impartiality) and context-specific obligations [9].
